# Increased miR-142-3p Expression Might Explain Reduced Regulatory T Cell Function in Granulomatosis With Polyangiitis

**DOI:** 10.3389/fimmu.2019.02170

**Published:** 2019-09-12

**Authors:** Gerjan J. Dekkema, Theo Bijma, Pytrick G. Jellema, Anke Van Den Berg, Bart-Jan Kroesen, Coen A. Stegeman, Peter Heeringa, Wayel H. Abdulahad, Jan-Stephan Sanders

**Affiliations:** ^1^Department of Pathology and Medical Biology, University Medical Center Groningen, University of Groningen, Groningen, Netherlands; ^2^Department of Internal Medicine, Division of Nephrology, University Medical Center Groningen, Groningen, Netherlands; ^3^Medical Immunology Laboratory, Department of Laboratory Medicine, University Medical Center Groningen, Groningen, Netherlands; ^4^Department of Rheumatology and Clinical Immunology, University Medical Center Groningen, University of Groningen, Groningen, Netherlands

**Keywords:** ANCA—associated vasculitis, Treg—regulatory T cell, auto immune, microRNA, suppressive function

## Abstract

**Objectives:** Regulatory T cells (Tregs) are frequently functionally impaired in patients with granulomatosis with polyangiitis (GPA). However, the mechanism underlying their impaired function is unknown. Here, we hypothesized that Treg dysfunction in GPA is due to altered microRNA (miRNA) expression.

**Methods:** RNA isolated from FACS-sorted memory (_M_) Tregs (CD4^+^CD45RO^+^CD25^+^CD127^−^) of 8 healthy controls (HCs) and 8 GPA patients without treatment was subjected to miRNA microarray analysis. Five differentially expressed miRNAs were validated in a larger cohort by reverse transcriptase quantitative polymerase chain reaction (RT-qPCR). An miRNA target gene database search revealed targets that were tested with RT-qPCR in _M_Tregs from patients and HCs. cAMP levels were measured using flow cytometry.

**Results:** Microarray analysis revealed 19 differentially expressed miRNAs, of which miR-142-3p was confirmed to be significantly upregulated in _M_Tregs from GPA patients compared to those from HCs (1.9-fold, *p* = 0.03). *In vitro* overexpression of miR-142-3p lowered the suppressive capacity of _M_Tregs (2.1-fold, *p* = 0.03), and miR-142-3p expression correlated negatively with the suppressive capacity (rho = −0.446, *p* = 0.04). Overexpression of miR-142-3p significantly decreased cAMP levels (*p* = 0.02) and tended to decrease the mRNA levels of a predicted target gene, adenylate cyclase 9 (ADCY9; *p* = 0.06). In comparison to those from HCs, _M_Tregs from GPA patients had lower ADCY9 mRNA levels (2-fold, *p* = 0.008) and produced significantly less cAMP after stimulation. Importantly, induction of cAMP production in miR-142-3p overexpressed _M_Tregs by forskolin restored their suppressive function *in vitro*.

**Conclusion:** Overexpression of miR-142-3p in _M_Tregs from GPA patients might cause functional impairment by targeting ADCY9, which leads to the suppression of cAMP production.

## Introduction

Antineutrophil cytoplasmic autoantibody (ANCA)-associated vasculitides (AAV) constitute a heterogeneous group of autoimmune syndromes characterized by pauci-immune necrotizing inflammation of small- to medium-sized blood vessels (1). These vasculitides of unknown etiology are predominantly associated with the presence of ANCAs directed against either proteinase-3 (PR3) or myeloperoxidase (MPO) (2). Based on the presence of specific ANCAs and clinical symptoms, AAV can be subdivided into microscopic polyangiitis (MPA), eosinophilic granulomatosis with polyangiitis (EGPA) and granulomatosis with polyangiitis (GPA), which is predominantly associated with the presence of PR3-ANCAs (1).

Although current immunotherapeutic strategies underscore the crucial role of B cells in GPA pathogenesis, several observations support the involvement of T cells in this disease. The presence of abundant T cell infiltrates in GPA lesions, persistent T cell activation with imbalances in circulating CD4^+^T cell subsets and the induction of remission by T cell-targeted therapies highlight the important role of T cell-mediated responses in GPA (3–9). Similar to those with various other autoimmune diseases, patients with GPA have impaired regulatory T cell (Treg) function (10–13). *In vitro* experiments have shown that circulating Tregs from GPA patients have a reduced ability to suppress the proliferation of activated effector cells (14–16). However, the exact mechanisms that contribute to the functional impairment of Tregs in GPA are currently unknown.

microRNAs (miRNAs) are single-stranded, noncoding RNA molecules of 19–22 nucleotides that regulate gene expression at the posttranscriptional level by binding complementary regions in the 3′ UTR of target messenger RNA (mRNA), leading to the degradation or translational inhibition of target mRNA (17). In recent years, many studies have identified a large number of miRNAs involved in the regulation of various T cell functions (17–19) and differential expression in T cells and Tregs is associated with T cell-mediated autoimmune diseases such as systemic lupus erythematosus (SLE), rheumatoid arthritis (RA), psoriasis and ulcerative colitis (20–24). For example, reduced upregulation of miRNA-146a after T cell activation, was observed in patients with RA compared to healthy controls. This diminished upregulation of miR-146a facilitated a proinflammatory phenotype of Tregs by increased levels of STAT1, a direct target of miR-146a (23). To date, it is unknown whether miRNAs are differentially expressed in Tregs of GPA patients and whether specific miRNAs are linked to the observed impaired suppressive function of these Tregs. In the current study, we hypothesized that differentially expressed miRNAs underlie the diminished suppressive function of Tregs in GPA.

Since the expanded Treg population in the peripheral blood of GPA patients is confined to memory cells (7), we examined the differential miRNA expression profile in sorted _M_Tregs, effector memory and naïve T cells from GPA patients.

## Subjects and Methods

### Subjects

Patients diagnosed with GPA based on the Chapel Hill Consensus classification and were PR3-ANCA positive were recruited (25). All included patients were in clinical remission with a Birmingham Vasculitis Activity Score (BVAS) of zero (26).

The inception cohort, containing eight patients with GPA and eight age- and sex-matched healthy controls, was selected for microarray-based miRNA expression profiling. Twenty-three patients and 23 healthy controls, including the patients selected for microarray analysis, were included in the validation cohort. Patient characteristics are shown in Table 1. This study was approved by the local Medical Ethics Committee (METC2010/057), and informed consent was obtained from all participants. The study was performed in accordance with the declaration of Helsinki.

**Table 1:** Patient characteristics.

	**miRNA inception cohort**		**miRNA validation cohort**		**cAMP cohort**	
	**Patients in remission**	**Healthy controls**	**Patients in remission**	**Healthy controls**	**Patients in remission**	**Healthy controls**
**Basic characteristics**
Number (*n*)	8	8	23	23	10	10
Median age (years)	55.7 (49.6–62.5)	53.9 (46.0–58.5)	53.1 (46.0–62.5)	54.0 (46.3–64.5)	57.5 (56.0–64.5)	58.6 (53.1–63.3)
Male, *n* (%)	5 (62.5%)	5 (62.5%)	11 (47.8%)	13 (56.5%)	4 (40%)	4 (40%)
**Disease Characteristics**
BVAS	0	–	0	–	0	–
Time after diagnosis (months)	168 (102–216)	–	149 (82–201)	–		–
Patients with relapse, *n* (%)	5 (62.5%)	–	12 (52.2%)	–	6 (60%)	–
Number of relapses, *n*	1 (0–3)	–	1 (0–2)	–	2 (0–3)	–
**Laboratory findings**
PR3-ANCA titer	40 (0–80)	–	40 (0–80)	–	80 (0–160)	–
Leucocytes (× 10^9^/l)	6.0 (4.8–7.0)	–	6.0 (5.3–6.9)	–	6.3 (5.1–7.4)	–
CRP (mg/l)	3 (1–6)	–	3 (1–4)	–	3 (2–6)	–
Current immunosuppressive treatment	1	0	3	0	0	0
Cyclophosphamide	0		0			
Azathioprine	1		1			
Prednisolone	0		2			

### Sample Preparation and Treg Cell Sorting

**Figure 1:**
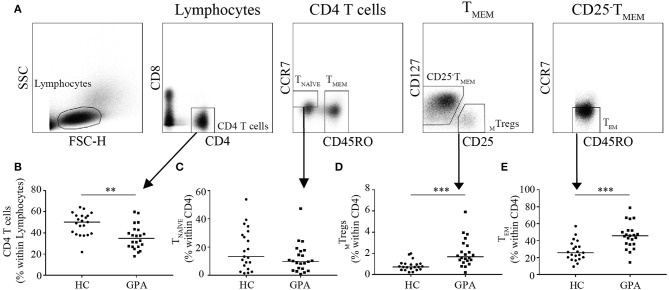
Frequencies of CD4^+^T cells and CD4^+^T cell subsets in healthy controls (HC) and GPA patients in remission (GPA). Representative FACS plots of sorted CD4^+^T cell subsets **(A)**. CD4^+^T cell frequencies were lower in GPA than in HC **(B)**. Within the CD4^+^T cell population, no differences were observed in naïve T (T_NAVE_) cells (CD4^+^CD45RO^−^CCR7^−^) **(C)**, whereas the _M_Treg (CD4^+^CD127^−^CD25^High^CD45RO^+^) frequency was higher in GPA than in HC **(D)**. A significantly higher frequency of CD4^+^T effector memory (T_EM_) cells (CD4^+^CD45RO^+^CCR7^−^) was observed in GPA than in HC **(E)**. **P = 0.01–0.001, ***P ≤ 0.001.

Peripheral blood was collected, and peripheral blood mononuclear cells (PBMCs) were isolated using density-gradient centrifugation on Lymphoprep (Axis-Shield, Oslo, Norway). Isolated PBMCs were stained with anti-CD8-AF700 (Affymetrix, San Diego, CA, USA); anti-CD4-eF450 (Thermo Fisher Scientific, Breda, The Netherlands); and anti-CD45RO-FITC, anti-CD127-AF647, anti-CD25-PE, and anti-CCR7-PE-CY7 (BD Biosciences, Breda, The Netherlands) and sorted using FACS (MoFlo Astrios, Beckman Coulter, Woerden, The Netherlands). Memory Tregs (_M_Tregs) (CD4^+^CD8^−^CD45RO^+^CD127^−^CD25^high^), naïve T (T_NAÏVE_) cells (CD4^+^CD8^−^CD45RO^−^CCR7^+^), and effector memory T (T_EM_) cells (CD4^+^CD8^−^CD45RO^+^CCR7^−^) were sorted (Figure 1A).

The purity of the sorted populations, was >95% for all samples. Samples were subsequently lysed using QIAzol lysis reagent (Qiagen, Venlo, The Netherlands) and stored at −80°C.

### RNA Isolation

Total RNA was extracted using the miRNeasy Micro Kit (Qiagen) according to the manufacturer's instructions. After isolation, RNA samples were further purified using Micro Bio-Spin columns (Bio-Rad, Veenendaal, The Netherlands).

### miRNA Microarray

Total RNA was hybridized to an Agilent G3 unrestricted miRNA microarray with an 8 × 60 K format (G4872-070156, based on miRBase Release 21.0) and scanned using an Agilent scanner according to the manufacturer's instructions (Agilent Technologies, Santa Clara, CA, USA). Array image data were extracted using Agilent Feature Extraction software (version 10.7). Data analysis was performed using GeneSpring software version 14.8. Raw data were normalized using the 95-percentile shift method. Control probes were excluded from the analysis. miRNAs with a signal intensity that reached >40% of the maximum signal in all samples of at least 1 cell type were considered for statistical analysis (two-way ANOVA with multiple sampling correction). A total of 19 miRNAs were differentially expressed, of which five miRNAs were selected for validation. This selection of the miRNAs for validation was based on availability of commercial assays (miR-6068, miR-4516 were not available at time of validation) and expression levels above detection limits of qPCR (miR-148a-3p, miR-27b-3p, miR-361-3p were expressed at low levels). The remaining 14 miRNAs were ranked based on both expression level and fold change between patients and healthy controls, and five miRNAs were selected for further validation.

### RT-qPCR

miRNA and gene expression levels were determined by RT-qPCR. For miRNA assays, total RNA was reverse transcribed using the TaqMan MicroRNA Reverse Transcription kit in a multiplex RT approach in combination with TaqMan MicroRNA Assays (both Thermo Fisher Scientific) for hsa-Let-7g-5p (#002282), hsa-miR-20a-5p (#000580), hsa-miR-26a-5p (#000405), hsa-miR-142-3p (#000464), hsa-miR-146b-5p (#001097), and RNU48 (#001006).

For gene expression assays, random hexamer reverse transcription of total RNA was performed using the High-Capacity cDNA Reverse Transcription Kit (Thermo Fisher Scientific) according to the manufacturer's instructions.

qPCR was performed on a ViiA7 Real-Time PCR System (Thermo Fisher Scientific) using qPCR MasterMix Plus (Eurogentec, Liege, Belgium) and TaqMan MicroRNA Assays for the miRs and TaqMan Gene Assays for human adenylate cyclase 9 (ADCY9) (Hs00181599_m1) and GAPDH (Hs02786624_g1) (Thermo Fisher Scientific). Mean threshold cycle (C_t_) values for all genes were quantified using QuantStudio Real-Time PCR software (Thermo Fisher Scientific). The expression levels of miRs relative to RNU48 and of ADCY9 relative to GAPDH were calculated using the 2^−Δ*Ct*^ method.

### miRNA Transfection and Suppression Assay

For the suppression assay, healthy control _M_Tregs and T responder (T_RESP_) cells (CD4^+^CD127^+^CD25^−^) were sorted. Sorted _M_Tregs were expanded using anti-CD3/CD28 Dynabeads and 200 International Units (IU)/ml IL-2 (Peprotech, Rocky Hill, USA) according to the manufacturer's protocol (Thermo Fisher Scientific) in RPMI1640 (Lonza, Breda, The Netherlands) supplemented with 10% human pooled serum (HPS) and 60 μg/ml gentamycin sulfate (Lonza).

Expanded _M_Tregs were transiently transfected with miRNA mimic scrambled control (SCR) or miRNA mimic hsa-miR-142-3p (MIM-142-3p) (Thermo Fisher Scientific) using the Nucleofector I system and the Human T cell Nucleofector kit (Lonza) according to the manufacturers' instructions. The Nucleofector program T-23 was used to transfect 50 nM mimic per 1.10^6^ cells. Transfected _M_Tregs were incubated overnight. _M_Tregs were harvested and live cells were sorted on a MoFlo XDP cell sorter (Beckman Coulter).

For the suppression assay, T_RESP_ (CD4^+^CD25^−^) cells were labeled with proliferation dye eFluor670 (1 μM; Thermo Fisher Scientific). T_RESP_ cells and _M_Tregs were cocultured at a 2:1 ratio and stimulated using anti-CD3/CD28 Dynabeads. After 3 days, the cells were stained with a viability dye, and T_RESP_ cell proliferation was analyzed on a BD LSRII flow cytometer. For cyclic adenosine monophosphate (cAMP) measurements (see below), transfected _M_Tregs were stimulated for 2 days with anti-CD3/CD28 Dynabeads.

### Stability of miRNA-142-3p Expression

To assess miR-142-3p expression levels in _M_Tregs after activation, sorted cells were stimulated for 24, 48, or 72 h with anti-CD3/CD28 Dynabeads and harvested for RNA isolation. Moreover, miR-142-3p expression levels in sorted unstimulated _M_Tregs and naïve _(N)_Tregs (CD4^+^CD8^−^CD45RO^−^CD127^−^CD25^+^) were determined.

### cAMP Production and FoxP3 Expression

PBMCs from 10 PR3-positive GPA patients and 10 matched HCs (cAMP cohort, Table 1) were stimulated with anti-CD3/anti-CD28 Dynabeads. Intracellular cAMP levels in _M_Tregs were assessed by flow cytometry. First, cells were fixed and permeabilized using a FoxP3 Fixation and Permeabilization kit (eBioscience) according to the manufacturer's protocol. Next, cells were stained with an unconjugated mouse-anti-cAMP antibody followed by a secondary goat-anti-mouse-PE antibody (Abcam, Cambridge, UK). _M_Tregs were stained using anti-CD3-PerCP, anti-CD4-eFluor450, anti-CD45RO-FITC, and anti-FoxP3-APC (eBioscience) and analyzed on a BD LSRII flow cytometer (Becton-Dickinson). Data were analyzed using Kaluza software (V1.5a, Beckman Coulter), and _M_Tregs were defined as CD3^+^CD4^+^CD45RO^+^FoxP3^high^.

### Forskolin Treatment of miR-142-3p Overexpressed _M_Tregs

In order to assess if cAMP elevating agent could restore Treg function, miR-142-3p overexpressed _M_Tregs were cultured in the presence and absence of 0.1 μM Forskolin (Sigma Aldrich, Saint Louis, Missouri, USA). Next, intracellular cAMP levels and suppressive capacity were assessed at 48 and 72 h, respectively.

### Statistical Analysis

Statistical analyses were performed using GraphPad Prism version 7 for Windows (GraphPad Software, San Diego, California, USA). Data were tested for normality using the D'Agostino-Pearson normality test; normally distributed data were analyzed for significant differences by Student's *T*-test. A *p*-value <0.05 was considered significant. As miRNA levels, cAMP levels and the cAMP total area under the curve (AUC) were not normally distributed, differences between the groups were analyzed using the Mann–Whitney test.

## Results

### GPA Patients Have a Higher Percentage of Circulating _M_Tregs

To explore differences in the distribution of circulating CD4^+^T cell subsets, we determined the frequency of _M_Tregs (CD4^+^CD25^High^CD45RO^+^), T_EM_ (CD4^+^CD45RO^+^CCR7^−^), and T_NAÏVE_ (CD4^+^CD45RO^−^CCR7^+^) in GPA patients and healthy controls. Patients had a significantly lower percentage of circulating CD4^+^T cells than did healthy controls (35.0 vs. 50.2%; Figure 1B). Additionally, the percentage of circulating _M_Tregs was significantly increased in patients compared to healthy controls (1.7 vs. 0.7%; Figure 1D). The percentage of circulating T_EM_ was significantly higher in patients than in healthy controls (45.6 vs. 25.7%; Figure 1E). No significant differences were found in the relative frequency of T_NAÏVE_ (13.3 vs. 9.9%; Figure 1C).

### Increased miR-142-3p Levels in GPA _M_Tregs

We compared the miRNA expression profiles of _M_Tregs, T_EM_, and T_NAÏVE_ from healthy controls and GPA patients. Nineteen miRNAs were found to be differentially expressed, of which 17 were significantly upregulated and two were significantly downregulated in GPA patients (Table 2, Supplemental Table 1). Based on a combination of expression levels, fold change and the commercial availability of assays, the top-5 miRs were selected for validation (hsa-let-7g-5p, hsa-miR-20a-5p, hsa-miR-26a-5p, hsa-miR-142-3p, and hsa-miR-146b-5p).

**Table 2:** Differentially expressed miRNAs in _M_Tregs.

	**HC**		**GPA**		**Fold change**	***p*-value**
ha-miR-142-3p	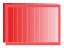	38.83	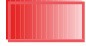	62.86	1.62	0.038
ha-let-7g-5p	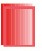	21.40	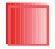	32.84	1.53	0.038
hsa-miR-26a-5p	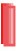	8.07	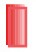	15.24	1.89	0.048
hsa-miR-20a-5p	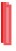	5.18	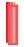	8.15	1.57	0.044
hsa-miR-146b-5p	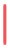	2.26	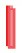	5.95	2.63	0.016
hsa-miR-4516	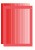	19.53	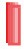	10.39	0.53	0.038
hsa-let-7f-5p	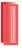	10.79	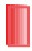	17.30	1.60	0.038
hsa-let-7a-5p	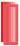	9.96	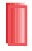	15.38	1.54	0.038
hsa-miR-19b-3p	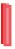	5.35	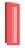	10.05	1.88	0.048
hsa-miR-103a-3p	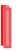	4.32	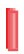	7.07	1.64	0.049
hsa-miR-107	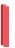	3.69	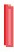	6.17	1.67	0.049
hsa-let-7d-5p	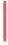	2.18	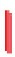	3.29	1.51	0.049
hsa-miR-17-5p	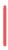	2.03	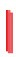	3.32	1.64	0.048
hsa-miR-6068	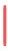	1.96	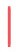	1.31	0.67	0.048
hsa-miR-20b-5p	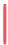	1.33	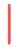	2.33	1.76	0.044
hsa-miR-30e-5p	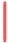	1.28	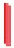	2.94	2.29	0.048
hsa-miR-361-3p		0.42		0.79	1.86	0.048
hsa-miR-27b-3p		0.14		0.54	3.76	0.046
hsa-miR-148a-3p		0.13		0.47	3.57	0.048

*miRNA which are increased in expression in GPA are highlighted in red, with reduced expression in GPA in blue*.

**Figure 2:**
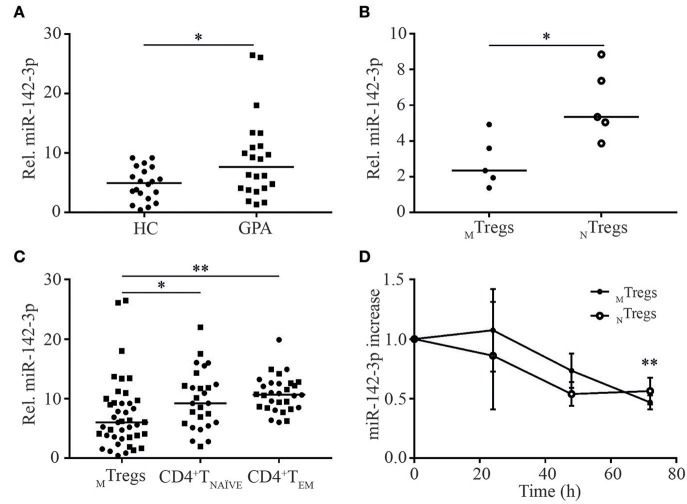
miR-142-3p is overexpressed in _M_Tregs from GPA patients in remission and is differentially regulated in CD4^+^T cell subsets. miR-142-3p levels were significantly higher in _M_Tregs from GPA patients (GPA) than in those from healthy controls (HC) **(A)**. miR-142-3p levels were significantly lower in _M_Tregs than in _N_Tregs **(B)** and in CD4^+^T_NAVE_ and CD4^+^T_EM_ cells from HC (circles) and GPA patients (squares) **(C)**. Stimulation of _M_Tregs and _N_Tregs with anti-CD3-CD28 Dynabeads led to significantly lower miR-142-3p levels over time (*n* = 5) **(D)**. *P = 0.05–0.01, **P = 0.01–0.001.

miR-142-3p expression levels were significantly higher in _M_Tregs from patients than in those from healthy controls (1.9-fold; Figure 2A). No differences were found in the expression of the other selected miRs in _M_Tregs or in the expression of all five miRs in T_NAÏVE_ and T_EM_ in the validation cohort (Supplemental Figure 1).

Interestingly, miR-142-3p expression was significantly lower in freshly isolated _M_Tregs than in naïve _(N)_Tregs (CD4^+^CD45RO^−^CD25^+^) from healthy controls (2.3-fold; Figure 2B) than in T_NAÏVE_ and T_EM_ cells from both healthy controls and patients (Figure 2C). The relatively low miR-142-3p expression levels in _M_Tregs might indicate that this miRNA is tightly regulated and low levels are necessary for efficient Treg function. Moreover, miR-142-3p levels in _M_Tregs and _N_Tregs from healthy controls tended to be lower after 48 h of activation (*p* = 0.11) and were significantly downregulated after 72 h of stimulation (2-fold, *p* = 0.006; Figure 2D).

Patient-related variables, such as age (*p* = 0.37), sex (*p* = 0.19), ANCA titer (*p* = 0.67), or immunosuppressive treatment, were not associated with miR-142-3p levels.

### Overexpression of miR-142-3p Reduces the Suppressive Capacity of _M_Tregs, Potentially *via* ADCY9

To determine whether increased levels of miR-142-3p influence Treg function, _M_Tregs from healthy controls were transfected with miR-142-3p mimic (MIM-142-3p) or scrambled control (SCR), and their ability to suppress T_RESP_ cell proliferation was measured *in vitro*.

**Figure 3:**
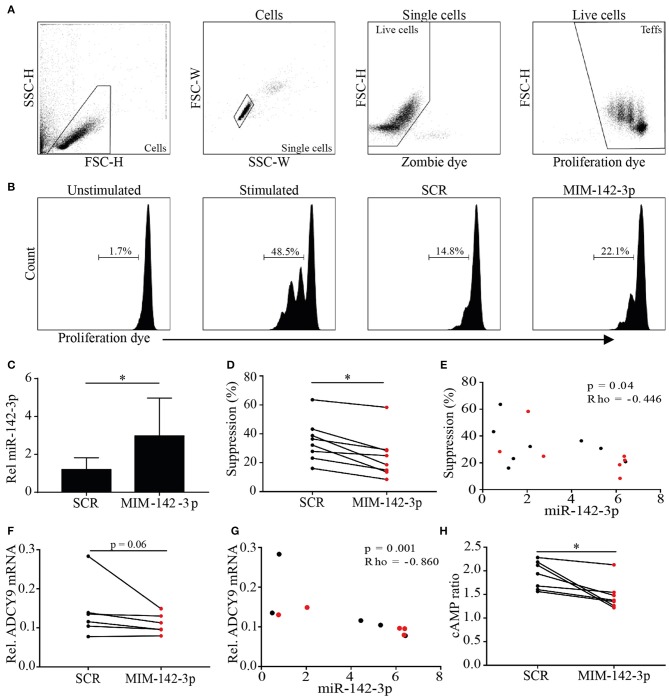
Overexpression of miR-142-3p reduces the suppressive capacity of _M_Tregs *in vitro*. _M_Tregs were transfected with scrambled (SCR) or miR-142-3p mimic (MIM-142-3p). Live _M_Tregs were sorted and cocultured with responder T (T_RESP_) cells; after 48 h of stimulation, T_RESP_ cell proliferation was determined **(A,B)**. Transfection with MIM-142-3p significantly increased miR-142-3p levels **(C)** and reduced the suppressive capacity of _M_Tregs **(D)**. miR-142-3p levels correlated negatively with the suppressive capacity **(E)**. Transfection with MIM-142-3p further decreased ADCY9 mRNA levels **(F)** and correlated negatively with miR-142-3p levels **(G)**. Additionally, cAMP levels were significantly lower in MIM-142-3p-transfected _M_Tregs (red) than in SCR-transfected _M_Tregs (black) **(H)**. *p = 0.05–0.01.

miR-142-3p levels were significantly increased in _M_Tregs transfected with MIM-142-3p compared to those transfected with SCR (2.4-fold, *p* = 0.03; Figure 3C). Both SCR- and MIM-142-3p-transfected Tregs were able to suppress T_RESP_ cell proliferation (Figures 3A,B). However, the suppressive capacity was significantly reduced in MIM-142-3p-transfected Tregs (2.1-fold, *p* = 0.03; Figure 3D). Importantly, we observed that miR-142-3p levels correlated negatively with the degree of suppression (rho = −0.446, *p* = 0.04; Figure 3E).

To explain the observed reduced suppressive capacity caused by miR-142-3p overexpression in Tregs, we searched for targets of miR-142-3p. Huang et al. reported that miR-142-3p overexpression in mouse Tregs reduces their suppressive capacity by targeting adenylate cyclase 9 (ADCY9). TargetScan version 7.1 predicted that ADCY9 is a highly conserved target of miR-142-3p, and human ADCY9 was confirmed as potential target (miRMap, TargetMiner). ADCY9 is a membrane-bound enzyme that catalyzes the conversion of adenosine triphosphate (ATP) into cAMP, which initiates suppression after delivery into effector T cells. Here, we hypothesized that miR-142-3p overexpression diminishes the Treg-mediated downregulation of ADCY9-induced cAMP production.

To assess whether miR-142-3p expression in Tregs influences ADCY9 and cAMP levels, ADCY9 and cAMP levels were measured after MIM-142-3p or SCR transfection. The overexpression of miR-142-3p was associated with decreased ADCY9 mRNA levels (1.3-fold, *p* = 0.06; Figure 3F). Importantly, miR-142-3p levels were significantly negatively correlated with ADCY9 mRNA levels (rho = −0.890, *p* = 0.001; Figure 3G). Additionally, after 48 h of stimulation, miR-142-3p overexpression led to significantly lower cAMP levels (1.4-fold, *p* = 0.02; Figure 3H).

### Decreased ADCY9 mRNA and cAMP Levels in _M_Tregs From GPA Patients

**Figure 4:**
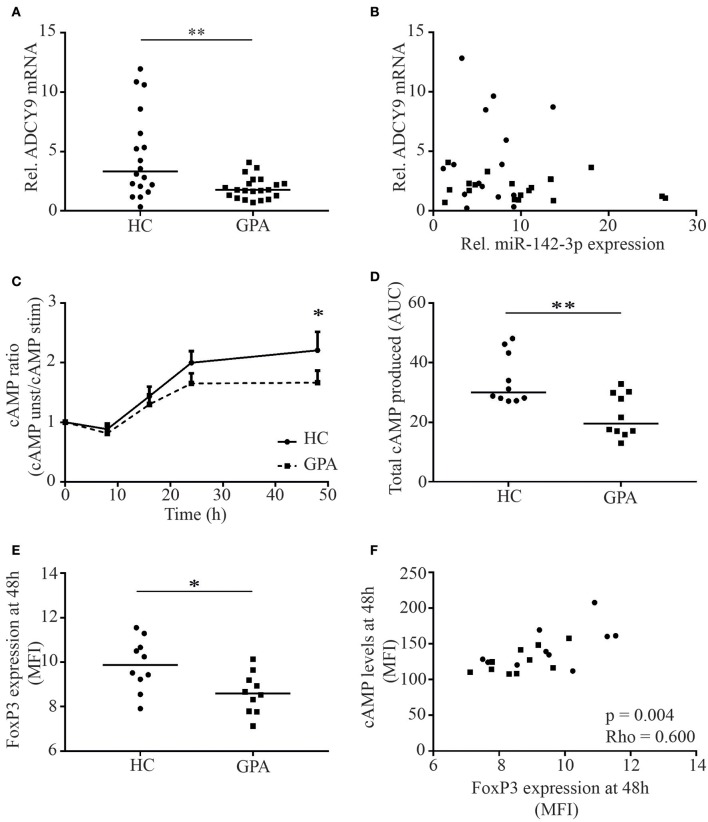
_M_Treg ADCY9 mRNA and cAMP levels are reduced in patients with GPA. ADCY9 mRNA levels were significantly lower in patients in remission (GPA) **(A)**, but mRNA levels of ADCY9 did not correlate with miR-142-3p expression **(B)**. In the cAMP cohort, cAMP levels were measured in _M_Tregs upon stimulation. cAMP levels in _M_Tregs were higher in healthy controls (HC) after 48 h stimulation (*n* = 10) than in GPA (*n* = 10) **(C)**, and total cAMP production was significantly higher in HC than in GPA **(D)**. Besides cAMP, FoxP3 expression was also higher in HC after stimulation **(E)** and FoxP3 levels correlated with cAMP **(F)**. *p = 0.05–0.01, **p = 0.01–0.001.

To provide further support for the role of ADCY9 in GPA, we assessed ADCY9 mRNA levels in _M_Tregs from GPA patients and healthy controls. ADCY9 mRNA levels were significantly lower in patients than in healthy controls (3.3 vs. 1.8, *p* = 0.008; Figure 4A). However, no correlation between miR-142-3p expression and ADCY9 mRNA levels was found (Figure 4B).

As ADCY9 mRNA levels were reduced in GPA, we next investigated whether this affected cAMP levels in stimulated _M_Tregs from GPA patients. cAMP levels were comparable at baseline but were significantly higher in _M_Tregs from healthy controls after 48 h of stimulation (MFI: 149 vs. 121, *p* = 0.042; data not shown). Moreover, total cAMP at 48 h after stimulation was significantly higher in _M_Tregs from healthy controls than in those from GPA patients (ratio: 2.4 vs. 1.9, *p* = 0.03; Figure 4C). In addition, the total amount of cAMP produced over 48 h of stimulation, as determined by the AUC, was significantly higher in _M_Tregs of healthy controls (34.2 vs. 22.3, *p* = 0.003; Figure 4D). In line with cAMP, FoxP3 levels were significantly higher in _M_Tregs from healthy controls than in those from patients (MFI: 9.6 vs. 8.5, *p* = 0.05; Figure 4E) and correlated strongly with cAMP levels (*p* = 0.003, rho = 0.600; Figure 4F) and total cAMP produced (*p* = 0.003, rho = 0.577).

### cAMP Elevating Therapy Restores _M_Treg Function *in vitro*

**Figure 5:**
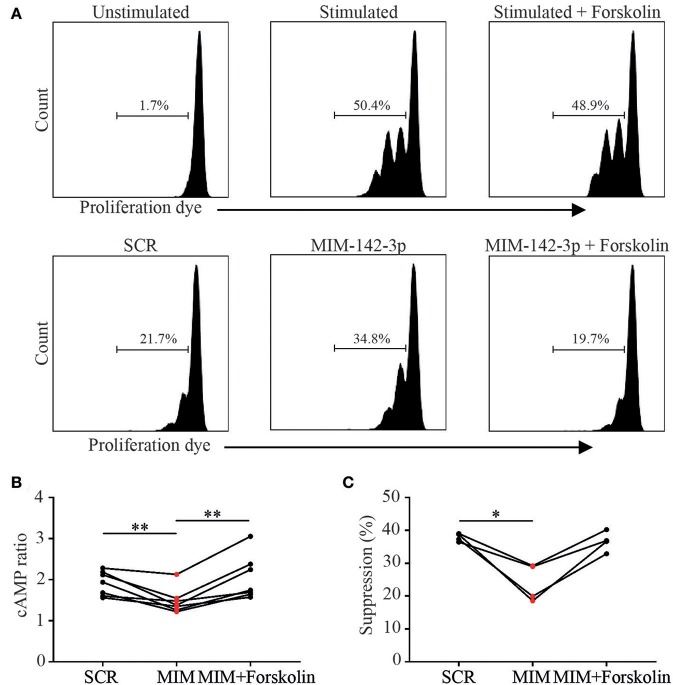
cAMP elevating agent Forskolin increases Treg function via the increase of cAMP. As proof of principle we tried to restore Treg function after miR-142-3p overexpression using cAMP elevating agent Forskolin. _M_Tregs were transfected with scrambled (SCR) or miR-142-3p mimic (MIM-142-3p). Live _M_Tregs were sorted and cocultured with responder T (T_RESP_) cells; after 48 h of stimulation, T_RESP_ cell proliferation was determined **(A)**. Upon treatment with Forskolin, cAMP levels increased significantly **(B)**. Moreover, forskolin treatment tended to restore suppressive function in miR-142-3p overexpressed _M_Tregs **(C)**.*p = 0.05–0.01, **p = 0.01–0.001.

We next aimed to restore the suppressive function of miR-142-3p overexpressed _M_Treg derived from healthy controls, by enhancing their intracellular cAMP levels *in vitro*. To this end, miR-142-3p overexpressed _M_Tregs were treated with cAMP elevating agent, Forskolin. Indeed, upon treatment with Forskolin, intracellular cAMP levels was significantly increased in miR-142-3p transfected _M_Tregs (Figure 5B).

Importantly, Forskolin treatment tended to restore the suppressive function of miR-142-3p overexpressed _M_Tregs in comparison to the non-transfected control cells (*p* = 0.06). In addition, no direct effect of Forskolin on T_RESP_ proliferation was seen (Figures 5A,C). These results offer further proof that miR-142-3p overexpression inhibits cAMP/ADCY9 suppression and that the reduced Treg suppression, induced by miR-142-3p overexpression, could be restored by Forskolin.

## Discussion

**Figure 6:**
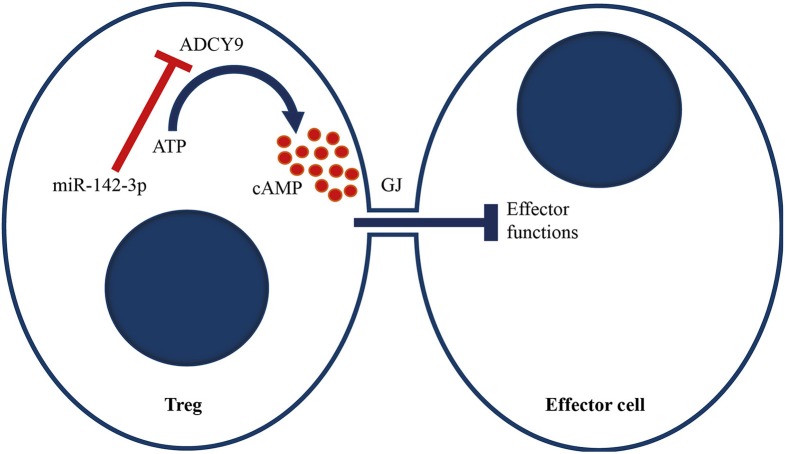
Proposed model of miR-142-3p, which targets the adenylyl cyclase 9/cAMP-mediated suppression of Tregs. We propose that miR-142-3p overexpression in GPA patients reduces ADCY9 levels, which leads to less conversion of ATP to cAMP upon stimulation. cAMP can be transferred to effector cells via a Gap junction (GJ). Upon transfection, cAMP levels in effector cells increase, which induces metabolic disruption and inhibits IL-2 production and cell proliferation. Increased miR-142-3p levels therefore decrease Treg function.

In the present study, we hypothesized that miRNA dysregulation could underlie the reduced _M_Treg function in patients with GPA. We found a significant increase in the miR-142-3p expression level in _M_Tregs from GPA patients. *In vitro* overexpression of miR-142-3p in healthy control _M_Tregs was associated with lower ADCY9 mRNA levels, reduced cAMP levels and a reduction in the suppressive capacity of Tregs. In addition, we found lower ADCY9 mRNA and cAMP levels in _M_Tregs from GPA patients than in those from healthy controls. Based on these findings, we conclude that miR-142-3p overexpression can decrease Treg function and may underlie the functional impairment seen in Tregs of patients with GPA. This impaired Treg function could be explained by the ADCY9-dependent downregulation of cAMP, a crucial axis that is prominently involved in the suppressive function of Tregs (Figure 6).

Numerous studies in autoimmune diseases, such as SLE, RA and psoriasis, have reported impaired Treg function (10–13). Additionally, the functional impairment of Tregs is well-established in GPA (14–16). However, the exact mechanisms underlying this reduced Treg suppressive capacity are not fully understood.

Tregs can suppress immune responses in several distinct manners, of which metabolic disruption via the transfer of cAMP to effector cells or the production of extracellular adenosine is considered a highly potent mechanism of suppression by Tregs (27–29). Klein et al. showed that blocking adenylyl cyclase (AC) activity reduced intracellular cAMP levels and the suppressive capacity of Tregs *in vitro* (30). This finding was supported by blocking AC activity in Tregs *in vivo*, which led to the inability to suppress graft-vs.-host disease in mice (30). Further evidence was provided by Bopp and coworkers, who demonstrated that blocking phosphodiesterase 4 (PDE4), a cAMP-degrading enzyme, in an allergic asthma mouse model led to increased cAMP levels and reduced airway hyperresponsiveness and inflammation (31).

Recent studies have shown that miR-142-3p can directly target ADCY9 mRNA and that its overexpression significantly reduces ADCY9 protein levels in mouse CD4^+^CD25^+^ Tregs (32, 33). In line with these results, we found that miR-142-3p overexpression in Tregs significantly reduced cAMP levels, which, in turn, led to a reduction in Treg-mediated suppression. Additionally, we found a significant trend toward a negative correlation between miR-142-3p levels and the suppressive capacity of Tregs, indicating that miR-142-3p influences Treg function via ADCY9 and cAMP.

Additionally, as proof of principle, cAMP elevating agent Forskolin was used to treat miR-142-3p overexpressed _M_Tregs. We showed that Forskolin was able to restore cAMP levels of miR-142-3p overexpressed _M_Tregs. Not only were cAMP levels restored, Forskolin also improved Treg mediated suppression. Our results are in line with a previous study which showed a significant increase in Treg function after treatment with cholera toxin, another cAMP elevating agent (34). Low dose Forskolin did not affect T_RESP_ alone. All in all, these data further supports the functional impact of miR-142-3p overexpression on ADCY9/cAMP mediated suppression and the ability to restore this functional deficit.

To date, the exact mechanism of miR-142-3p regulation is not fully understood and could be linked to intrinsic or extrinsic factors. Previously, a link between FoxP3 and miR-142-3p expression was identified. Overexpression of FoxP3 in CD4^+^CD25^−^T cells led to a substantial decrease in miR-142-3p levels (33). In accordance with this finding, we found that miR-142-3p expression was lower in _M_Tregs than in other FoxP3-negative CD4^+^T cell subsets. Moreover, we showed that upon activation of _M_Tregs, FoxP3 expression and cAMP levels increased, whereas miR-142-3p levels decreased significantly. Collectively, these data indicate that FoxP3 is an important factor in the regulation of miR-142-3p expression in _M_Tregs.

Previous studies have shown that Tregs can express different FoxP3 isoforms, of which the most common are FoxP3 full length, FoxP3 lacking exon 2 (FoxP3ΔE2), and FoxP3 lacking exons 2 and 7 (FoxP3ΔE2ΔE7) (35). The expression of FoxP3 isoforms other than the full-length form is associated with diminished Treg function (36). It has been reported that the proportion of FoxP3ΔE2-expressing Tregs is increased in AAV, including GPA, and correlates negatively with the degree of suppression (14). Since we demonstrate here that the suppressive capacity of Tregs is associated with miR-142-3p expression, one could speculate that FoxP3ΔE2-expressing Tregs have reduced suppressive capacity because of their diminished ability to suppress miR-142-3p expression. Clearly, further studies are required to investigate the potential links among FoxP3 isoform expression, miR-142-3p, and Treg function.

In addition to FoxP3 expression, immunosuppressive medication could also influence miR expression levels. A recent study showed that *in vitro* treatment of CD4^+^T cells with mycophenolate mofetil induced miR-142-3p expression (37). We did not detect a pronounced effect of treatment on miR-142-3p expression levels in our study samples. However, most of the included patients had not received immunosuppressive treatment at the time of sampling, so it would be difficult for us to detect such effects.

This study was cross-sectional; it would be interesting to study miR-142-3p levels in T cells from GPA patients in a longitudinal manner. Moreover, a previous study showed that some patients have normal Treg function *in vitro* (15). We also found that miR-142-3p levels were not higher in all patients than in healthy controls. Previous studies have also reported that not in all GPA patients Treg function is diminished. In our cohorts, we previously found that ~60–70% of GPA patients have Tregs with diminished suppressive function (15). This is in line with our finding that miR-142-3p is only increased in a subset of GPA patients. Additionally, it is conceivable that Tregs have additional defects not mediated by the miR-142-3p-ADCY9-cAMP axis.

In conclusion, increased expression of miR-142-3p in _M_Tregs from patients with GPA might underlie their functional impairment by modulating ADCY9-mediated cAMP production. Our results suggest that therapeutic interventions aiming to restore miR-142-3p and cAMP levels in Tregs present a novel approach to restore Treg function in GPA patients and potentially in those with other autoimmune diseases in which there is a functional defect in the Treg subset.

## Data Availability

The gene expression data was deposited in ArrayExpress - E-MTAB8282. All other data supporting the conclusions of this manuscript will be made available by the authors, without undue reservation, to any qualified researcher.

## Ethics Statement

Peripheral blood samples from patients and healthy controls were used for this study. This study was approved by the local Medical Ethics Committee of the university of Groningen/University medical center Groningen (METC number: METC2010/057), and informed consent was obtained from all participants. The study was performed in accordance with the declaration of Helsinki.

## Author Contributions

GD designed, performed experiments, did the data analysis, and wrote the manuscript. TB designed, performed experiments, and did the data analysis. PJ performed experiments. AV and B-JK assisted in the data analysis and designed the study. CS collected patient and control samples. PH and WA designed the study and contributed to the manuscript. J-SS had the initial idea and contributed to the manuscript.

### Conflict of Interest Statement

The authors declare that the research was conducted in the absence of any commercial or financial relationships that could be construed as a potential conflict of interest.

## Supplementary Material

Supplemental Figure 1 Validation of five differentially expressed miRs in the validation cohort. Relative expression of the microRNAs hsa-let-7g-5p **(A–C)**, hsa-miR-20a-5p **(D–F)**, hsa-miR-26a-5p **(G–I)**, hsa-miR-146b-5p **(J,L)**, and hsa-miR-142-3p **(M,N)** relative to RNU48. miRNAs were validated using RT-qPCR analysis of total RNA from FACS-sorted _M_Tregs **(A,D,G,J)**, T_NAÏVE_ cells **(B,E,H,K,M)**, and T_EM_ cells **(C,F,I,L,N)** from healthy controls (HC) and GPA patients in remission (REM). The relative expression of miR-142-3p compared to RNU48 in Tregs is depicted in Figure 1A.

Supplemental Table 1 Differentially expressed miRNAs in T_NAÏVE_ and T_EM_.
