# Supplementary figures and images for: Increased miR-142-3p Expression Might Explain Reduced Regulatory T Cell Function in Granulomatosis With Polyangiitis
Source: Front Immunol. 2019 Sep 12;10:2170. doi: 10.3389/fimmu.2019.02170 (PMC6751284; doi:10.3389/fimmu.2019.02170)

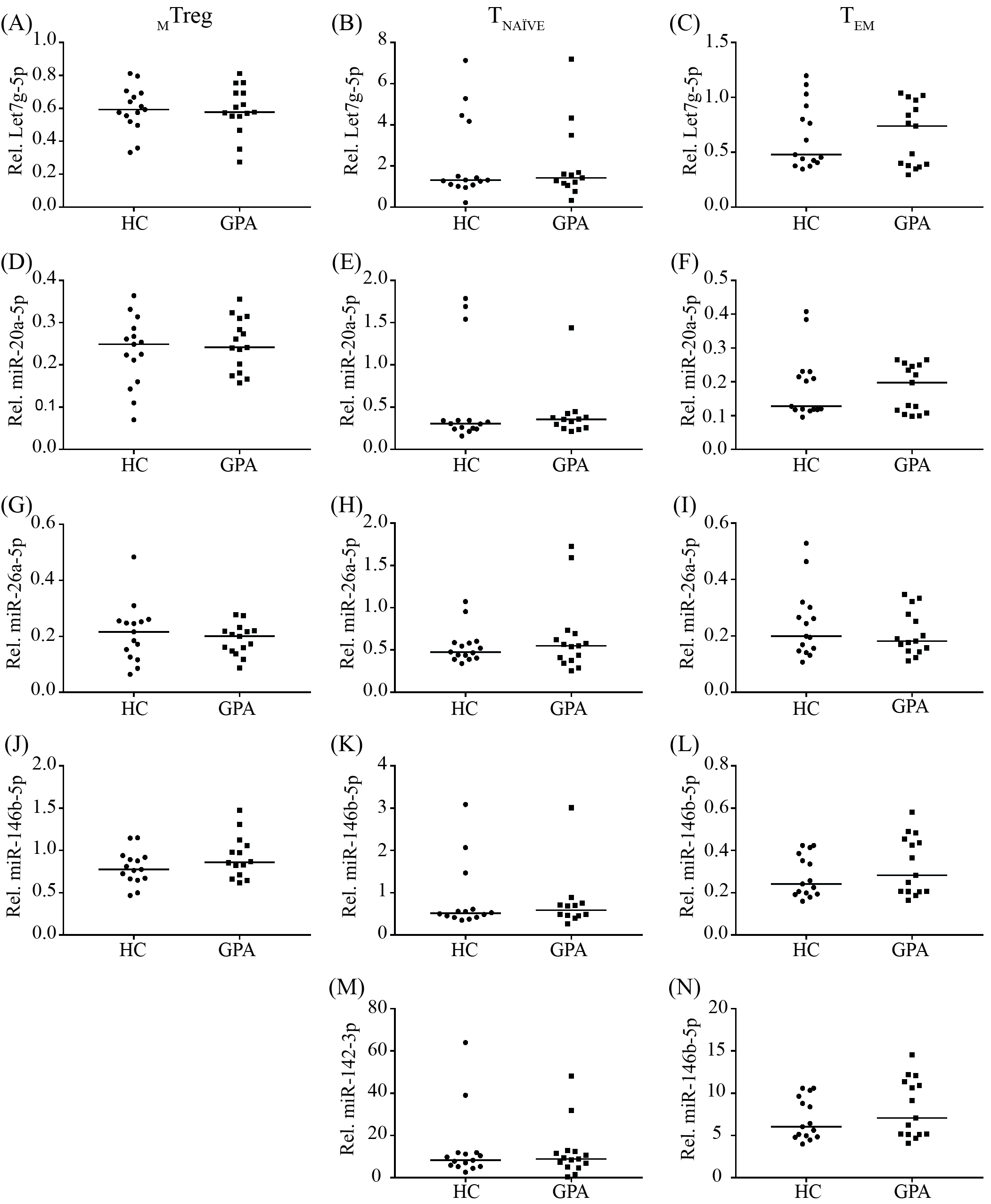

Supplement: Supplemental Figure 1 — Validation of five differentially expressed miRs in the validation cohort. Relative expression of the microRNAs hsa-let-7g-5p (A–C), hsa-miR-20a-5p (D–F), hsa-miR-26a-5p (G–I), hsa-miR-146b-5p (J,L), and hsa-miR-142-3p (M,N) relative to RNU48. miRNAs were validated using RT-qPCR analysis of total RNA from FACS-sorted MTregs (A,D,G,J), TNAÏVE cells (B,E,H,K,M), and TEM cells (C,F,I,L,N) from healthy controls (HC) and GPA patients in remission (REM). The relative expression of miR-142-3p compared to RNU48 in Tregs is depicted in Figure 1A. [file Image_1.JPEG]
