# Supplementary material for: Increased miR-142-3p Expression Might Explain Reduced Regulatory T Cell Function in Granulomatosis With Polyangiitis
Source: Front Immunol. 2019 Sep 12;10:2170. doi: 10.3389/fimmu.2019.02170 (PMC6751284; doi:10.3389/fimmu.2019.02170)
Supplement: Supplemental Table 1 — Differentially expressed miRNAs in TNAÏVE and TEM. [file Table_1.pdf]

Supplemental table 2: Differentially expressed miRNAs in T_{NAIVE} and T_{EM}

	$T_{NAÏVE}$					T_{EM}		
	HC	GPA	Fold change			HC	GPA	Fold change
hsa-miR-142-3p	46,79	96,36	2,06	0.038	hsa-miR-142-3p	38,31	134,36	3,51
hsa-let-7g-5p	45,99	56,04	1,22	0.038	hsa-let-7g-5p	31,53	54,47	1,73
hsa-miR-26a-5p	32,34	34,14	1,06	0.048	hsa-miR-26a-5p	16,03	25,72	1,60
hsa-miR-20a-5p	2,78	4,40	1,58	0.044	hsa-miR-20a-5p	1,40	3,20	2,29
hsa-miR-146b-5p	10,30	16,14	1,57	0.016	hsa-miR-146b-5p	5,47	13,77	2,51
hsa-miR-4516	8,04	6,60	0,82	0.038	hsa-miR-4516	9,98	6,95	0,70
hsa-let-7f-5p	19,20	22,29	1,16	0.038	hsa-let-7f-5p	14,36	23,06	1,61
hsa-let-7a-5p	21,88	23,63	1,08	0.038	hsa-let-7a-5p	15,24	21,74	1,43
hsa-miR-19b-3p	12,07	17,91	1,48	0.048	hsa-miR-19b-3p	6,46	15,45	2,39
hsa-miR-103a-3p	5,59	6,29	1,13	0.049	hsa-miR-103a-3p	5,33	7,45	1,40
hsa-miR-107	3,79	4,35	1,15	0.049	hsa-miR-107	4,03	5,96	1,48
hsa-let-7d-5p	3,40	3,75	1,10	0.049	hsa-let-7d-5p	2,79	3,57	1,28
hsa-miR-17-5p	3,65	4,73	1,29	0.048	hsa-miR-17-5p	2,41	3,93	1,63
hsa-miR-6068	1,47	1,31	0,89	0.048	hsa-miR-6068	1,50	1,02	0,68
hsa-miR-20b-5p	8,78	13,23	1,51	0.044	hsa-miR-20b-5p	5,30	10,98	2,07
hsa-miR-30e-5p	2,32	3,66	1,58	0.048	hsa-miR-30e-5p	1,47	4,08	2,77
hsa-miR-361-3p	0,96	0,94	0,98	0.048	hsa-miR-361-3p	0,70	1,01	1,44
hsa-miR-27b-3p	0,24	0,57	2,36	0.046	hsa-miR-27b-3p	0,17	0,75	4,46
hsa-miR-148a-3p	0,27	0,61	2,23	0.048	hsa-miR-148a-3p	0,17	0,79	4,76
